# New Insights Into PTBP3 in Human Cancers: Immune Cell Infiltration, TMB, MSI, PDCD1 and m6A Markers

**DOI:** 10.3389/fphar.2022.811338

**Published:** 2022-03-10

**Authors:** Zhen Fang, Peijuan Li, Han Li, Wei Chong, Leping Li, Liang Shang, Fei Li

**Affiliations:** ^1^ Department of General Surgery, Xuanwu Hospital, Capital Medical University, Beijing, China; ^2^ Emergency Department, The First Affiliated Hospital of Dalian Medical University, Dalian, China; ^3^ Department of General Surgery, Shandong Provincial Qianfoshan Hospital, The First Affiliated Hospital of Shandong First Medical University, Jinan, China; ^4^ Department of Gastrointestinal Surgery, Shandong Provincial Hospital Affiliated to Shandong First Medical University, Jinan, China

**Keywords:** PTBP3, pan-cancer, prognosis, immunotherapy, phosphorylation, m6A

## Abstract

Polypyrimidine tract binding protein 3 (PTBP3) plays a critical role in post-transcriptional regulation. The role of PTBP3 in various human tumours was explored and analysed in this study based on the Cancer Genome Atlas and Gene Expression Omnibus datasets. PTBP3 was highly expressed in most tumours, such as breast invasive carcinoma, colon adenocarcinoma and hepatocellular carcinoma. PTBP3 overexpression generally predicts poor overall survival and disease-free survival in patients with adrenocortical carcinoma, lung squamous cell carcinoma, and pancreatic adenocarcinoma. However, low PTBP3 expression predicts poor prognosis in kidney renal clear cell carcinoma. We also explored PTBP3 genetic alterations in different tumour tissues. The result found that the frequency of PTBP3 alteration (>4%) was the highest in uterine tumours with “mutation” as the primary type. Furthermore, we found a significant correlation between PTBP3 expression and tumour mutational burden and microsatellite instability in various human tumours, and found that PTBP3 expression was positively correlated with TMB in ACC, STAD, PAAD, LUAD, and SARC. Two enhanced phosphorylation levels of S30 and S426 in colon cancer, ovarian cancer, and uterine corpus endometrial carcinoma were found. Further analysis indicated that PTBP3 expression was positively correlated with the cancer-associated fibroblasts for most tumour types. This study also found a relationship between immune checkpoints and N6-methyladenosine-related markers and PTBP3 expression. Moreover, the “mRNA surveillance pathway” and “RNA degradation” were involved in the functional mechanisms of PTBP3. These results provide new insights for molecular studies, and integrative analysis provided a framework for determining the predictive, prognostic, and therapeutic relevance of PTBP3 in cancer patients.

## Introduction

Polypyrimidine tract binding protein 3 (PTBP3), also known as the regulator of differentiation (ROD) 1, is located on human chromosome 9q32. PTBP3 is a member of the polypyrimidine tract binding (PTB) protein family, which also includes PTBP1 and PTBP2 (Hou et al., 2019). The PTB family is known to bind to CU-rich elements in exon and intron regions (Marinescu et al., 2007). The PTB family binding of CU-rich sequences can also influence splice site selection by preventing the transition from exons to introns, or by obstructing the definition of exons and introns (Sharma et al., 2008; Ma et al., 2020).

Previous studies have indicated that PTBP3 is crucial in the complex process of tumorigenesis (Hou et al., 2018; Liang et al., 2018; Yang et al., 2018; Hou et al., 2019; Ma et al., 2020; Wu et al., 2020; Chen et al., 2022). For example, PTBP3 regulates lung cancer cell proliferation through the cell cycle and may be a potential target for lung cancer molecular therapy (Chen et al., 2022). PTBP3 can promote colorectal cell proliferation, migration, and invasion *in vitro* and tumour growth and metastasis *in vivo* by binding to the 5′UTR HIF-1α mRNA to enhance HIF-1α protein expression (Hou et al., 2019). PTBP3 overexpression also promotes pancreatic ductal adenocarcinoma proliferation *in vitro* and tumour growth *in vivo* (Ma et al., 2020). In addition, PTBP3 can regulate the expression of ZEB1 (epithelial-mesenchymal transition regulatory transcription factor) and promotes tumour cell invasive growth and metastasis in breast cancer (Hou et al., 2018). PTBP3 expression is positively correlated with lymph node metastasis and poor 5-year survival in patients with breast cancer (Hou et al., 2018). Therefore, these current findings imply that PTBP3 may be a new and promising tumour biomarker.

In this study, The Cancer Genome Atlas (TCGA) Project and Gene Expression Omnibus (GEO) databases were used to explore the expression profile of PTBP3 across various tumour types. The main investigated contents are as follows: gene expression profile, survival status, genetic alteration, protein phosphorylation, immune infiltration, N6-methyladenosine, and relevant cellular pathways. Comprehensive pan-cancer analysis aims to investigate the potential molecular mechanism of PTBP3 in the pathogenesis, clinical prognosis, and immunotherapy of various human tumours.

## Materials and Methods

### Gene Expression Analysis

We used Tumour Immune Estimation Resource, Version 2 (TIMER2, http://timer.cistrome.org/) to analyse the differences in PTBP3 expression in different tumour types and adjacent normal tissues. However, lymphoid neoplasm diffuse large B-cell (DLBC) lymphoma, brain lower grade glioma (LGG), ovarian serous cystadenocarcinoma (OV), testicular germ cell tumours (TGCT), and thymoma (THYM) had only tumour tissue but no adjacent normal tissue. The Gene Expression Profiling Interactive Analysis, version 2 (GEPIA2) website was used to analyse PTBP3 expression in DLBC, LGG, OV, TGCT, and THYM (Tang et al., 2019). UALCAN can provide protein expression analysis options using data from the Clinical Proteomic Tumour Analysis Consortium (CPTAC) dataset. We used the UALCAN website (http://ualcan.path.uab.edu/index.html) to analyse the protein expression of PTBP3 in breast cancer, colon cancer, uterine corpus endometrial carcinoma (UCEC), lung adenocarcinoma, and ovarian cancer. The GEPIA2 website was also used to analyse PTBP3 expression in different pathological stages of all TCGA cancers.

### Survival Prognosis Analysis

The GEPIA2 tool was used to obtain the overall survival (OS) and disease-free survival (DFS) significance map data across all TCGA tumours (Tang et al., 2019). The median was used as the expression threshold for splitting the high- and low-expression groups. Month units were selected for the plotting. The Cox proportional hazard ratio and 95% confidence interval information were used in the survival plot. The log-rank test was used for the hypothesis testing.

### Genetic Alteration Analysis

The cBioPortal tool was used to analyse the alteration frequency, mutation type, copy number alteration, and mutated site information across all TCGA tumours (Cerami et al., 2012; Gao et al., 2013). After logging in to the cBioPortal website, select “TCGA Pan Cancer Atlas Studies” to query the genetic alteration characteristics of PTBP3. We could observe results for alteration frequency, mutation type and copy number alterations (CNAs) in all TCGA tumours. The mutation site information of PTBP3 can be displayed through the “Mutations” module. At the same time, the 3D structure of the protein can also be obtained. The OS and DFS data for all TCGA cancer types were compared with or without PTBP3 genetic alterations.

### Immune Infiltration and N6-Methyladenosine-Related Makers Analysis

We analysed the association between PTBP3 expression and immune infiltrates (e.g., cancer associated fibroblast and neutrophil) across all TCGA tumours and the relationship between PTBP3 expression and N6-methyladenosine (m^6^A)-related markers (e.g., METTL3, KIAA1429, FTO, ZC3H13, YTHDF1/2/3, RBM15) using the TIMER2 tool. The relationship between PTBP3 expression and immune checkpoints (e.g., PD1, PDL1, CTLA4, CD274, LAG3, SIGLEC15, TIGIT) was explored using the HOME FOR RESEARCHERS website tool (https://www.aclbi.com/).

### PTBP3-Related Gene Enrichment Analysis

The STRING tool (https://string-db.org/) was used to construct the protein-protein interaction network (Szklarczyk et al., 2021). The main parameters are as follow: minimum required interaction score [“Low confidence (0.150)”], max number of interactors to show (“no more than 50 interactors” in the 1st shell), meaning of network edges (“evidence”), and active interaction sources (“experiments”). The GEPIA2 tool was used to obtain the top 100 PTBP3-related targeting genes based on the database of all TCGA tissues. Furthermore, we performed pairwise gene correlation analysis of PTBP3 and the selected genes. Pearson’s correlation analysis was also used to calculate the correlation coefficient, while the heatmap of the selected genes contained the partial correlation and *p*-value in the purity-adjusted Spearman’s rank correlation. The R package of “tidyr” and “ggplot2” were used to visualise the enrichment pathways. R software (R-4.0.2, 64-bit) was used in this study (https://www.r-project.org/), and statistical significance was set at *p* < 0.05 (Cui et al., 2020; Huo et al., 2021).

## Results

### Gene Expression Analysis Data

In this study, we have provided a comprehensive analysis of the role of PTBP3 (NM_001163788.4 for NP_001157260.1, Supplementary Figure S1) in humans. We analysed the expression of PTBP3 in different cell lines and normal tissues (Supplementary Figure S2). We found that PTBP3 expression in BeWo cells (human placental choriocarcinoma cells) was the highest in the Human Protein Atlas (HPA) datasets, followed by K-562 (chronic myelogenous leukaemia cells) and OE19 (human oesophageal cancer cells) (Supplementary Figure S2A). As shown in Supplementary Figure S2B, we collected data from the Consensus, HPA, Genotype-Tissue Expression (GTEx), and FANTOM5 (Function Annotation of the Mammalian Genome 5) datasets. This comparison showed high expression of PTBP3 in the oesophagus, tonsils, lymph nodes, and thymus. However, PTBP3 was expressed in all tissues (all consensus normalised expression values >1), and it showed low tissue specificity.

**FIGURE 1:**
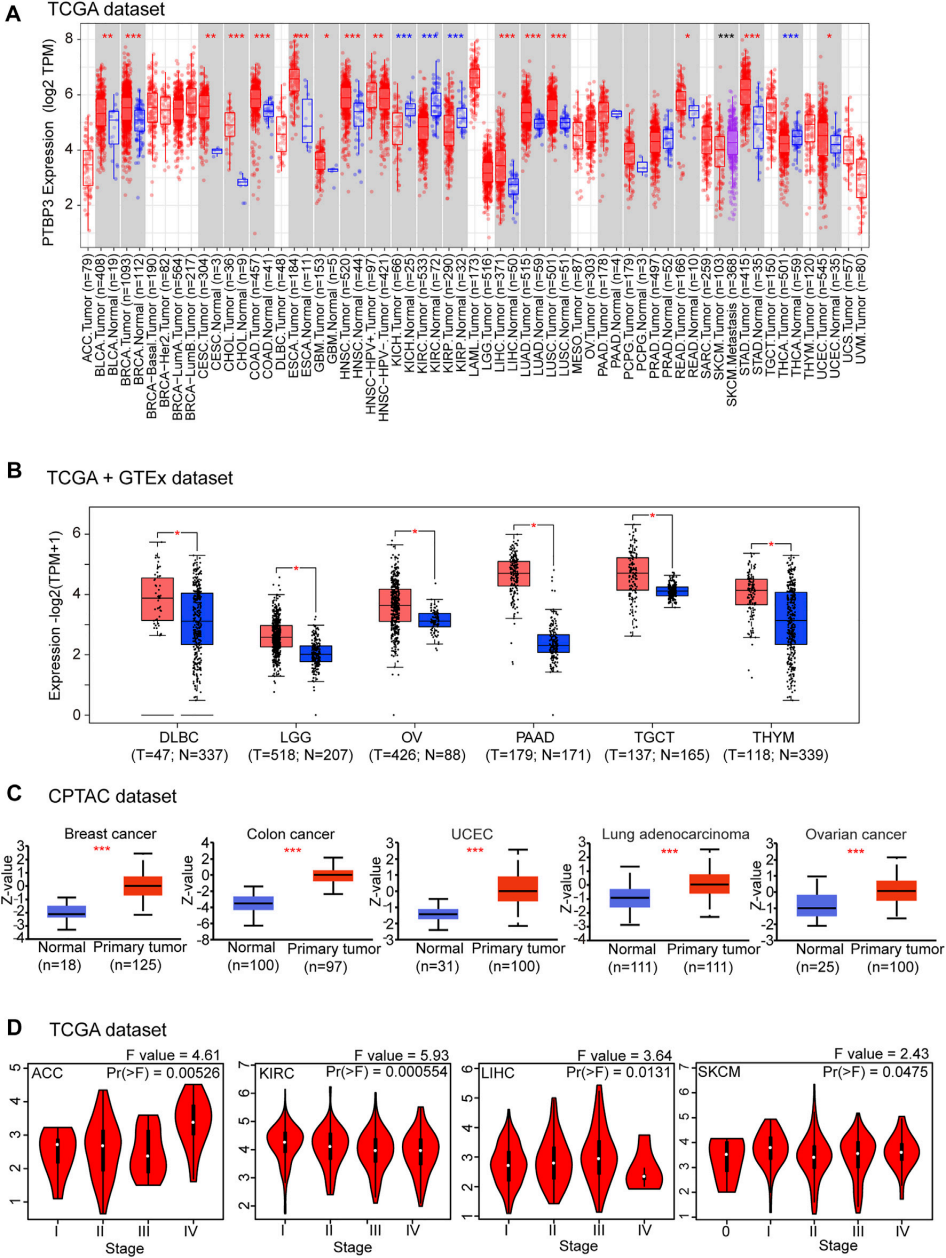
Expression and protein levels of PTBP3 in tumour and normal tissues. **(A)** The expression level of PTBP3 in different tumour types and adjacent normal tissues was visualised using TIMER2 (TCGA dataset). *p < 0.05; **p < 0.01; ***p < 0.001. **(B)** PTBP3 expression levels in DLBC, LGG, OV, TGCT, and THYM relative to normal tissues (TCGA + GTEx dataset). *p < 0.05. **(C)** The expression level of PTBP3 total protein between normal and primary tumour tissues of breast cancer, colon cancer, UCEC, lung adenocarcinoma, and ovarian cancer (CPTAC dataset). ***p < 0.001. **(D)** The main pathological stages (stages I, II, III, and IV) of ACC, KIRC, LIHC, and SKCM were analysed (TCGA dataset). TIMER2: Tumour Immune Estimation Resource, Version 2; DLBC: Lymphoid neoplasm diffuse large B-cell; LGG: Brain lower grade glioma; OV: Ovarian serous cystadenocarcinoma; TGCT: Testicular germ cell tumours; THYM: Thymoma; TCGA: The Cancer Genome Atlas; GTEx: Genotype-Tissue Expression; UCEC: Uterine corpus endometrial carcinoma; CPTAC: Clinical Proteomic Tumor Analysis Consortium; ACC: Adrenocortical carcinoma; KIRC: Kidney renal clear cell carcinoma; LIHC: Liver hepatocellular carcinoma; SKCM: Skin Cutaneous Melanoma.

Next, we used TIMER2 to explore the expression of PTBP3 in different tumour types in the TCGA repository. As shown in Figure 1A, the expression levels of PTBP3 in breast invasive carcinoma (BRCA), cervical squamous cell carcinoma and endocervical adenocarcinoma (CESC), cholangiocarcinoma (CHOL), colon adenocarcinoma (COAD), oesophageal carcinoma (ESCA), glioblastoma multiforme (GBM), head and neck squamous cell carcinoma (HNSC), liver hepatocellular carcinoma (LIHC), lung adenocarcinoma (LUAD), lung squamous cell carcinoma (LUSC), rectum adenocarcinoma (READ), stomach adenocarcinoma (STAD), and uterine corpus endometrial carcinoma (UCEC) (all *p* < 0.05) were higher than those in the corresponding adjacent normal tissues. Only a few tumour types showed no differential expression (e.g., pancreatic adenocarcinoma [PAAD], pheochromocytoma and paraganglioma [PCPG], prostate adenocarcinoma [PRAD]). In contrast, PTBP3 showed lower expression in the tumours of kidney chromophobe (KICH), kidney renal clear cell carcinoma (KIRC), kidney renal papillary cell carcinoma (KIRP), and thyroid carcinoma (THCA) (all *p* < 0.05) relative to the corresponding adjacent normal tissues.

After including the GTEx dataset, we further analysed the differences in PTBP3 expression between the tumour and normal tissues. We obtained significant difference for six tumour types (e.g., DLBC, LGG, OV, PAAD, TGCT, and THYM) (all *p* < 0.05) (Figure 1B). There were no significant differences in other tumours (e.g., adrenocortical carcinoma (ACC), bladder urothelial carcinoma (BLCA), acute myeloid leukaemia, PCPG, PRAD, sarcoma (SARC), and uterine carcinosarcoma (UCS) (Supplementary Figure S3). In general, we found that the expression of PTBP3 is elevated in most human tumours.

We also assessed the PTBP3 protein levels. The results of the National Cancer Institute’s CPTAC dataset showed that the total protein expression of PTBP3 was significantly higher in BRCA, COAD, UCEC, LUAD, and OV (all *p* < 0.05) than in normal tissues (Figure 1C). We further analysed the relationship between PTBP3 expression and tumour pathological staging and found stage-specific expressional changes in PTBP3 expression (e.g., ACC, KIRC, LIHC, Skin Cutaneous Melanoma (SKCM)) (Figure 1D).

### Survival Analysis Data

**FIGURE 2:**
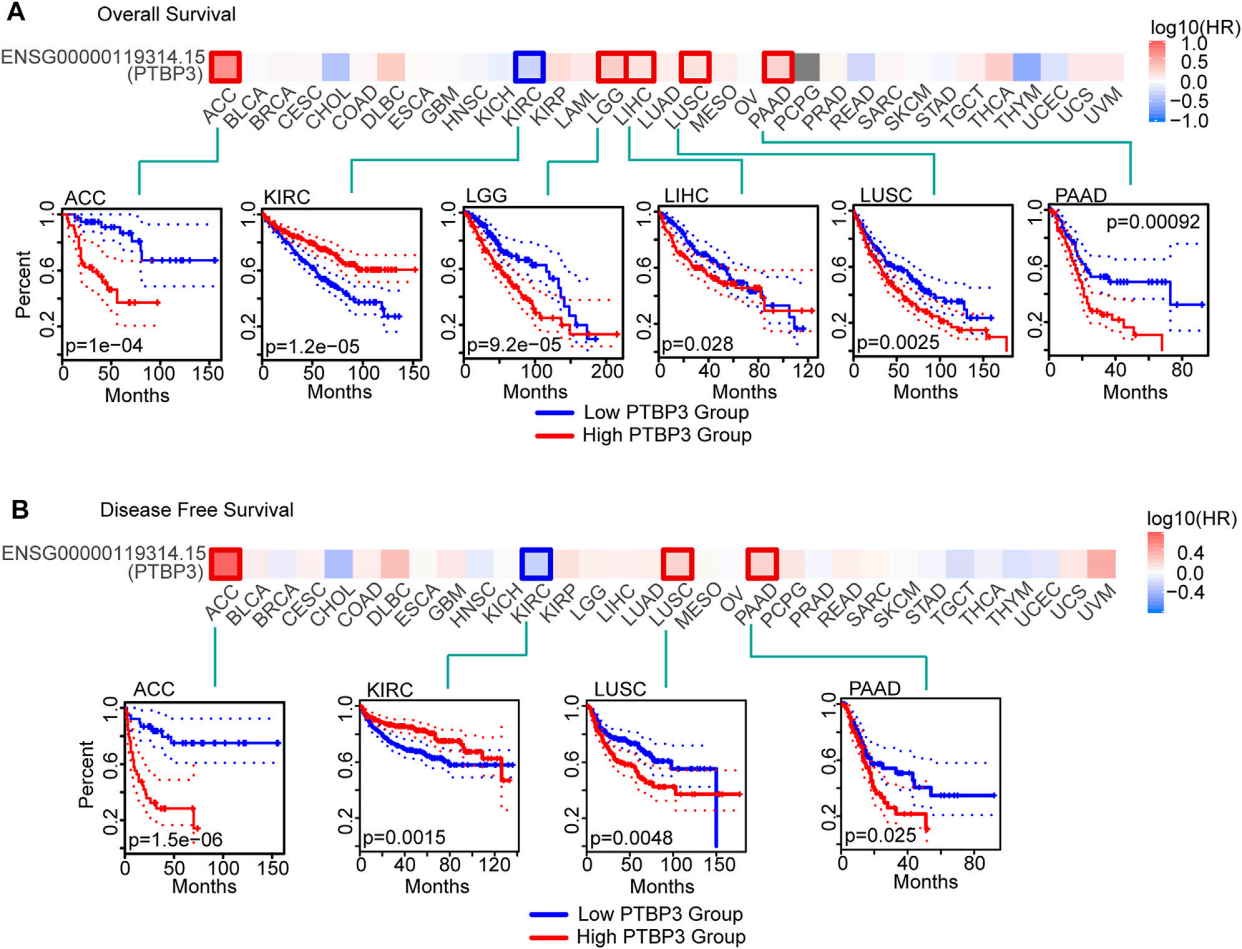
Relationship between PTBP3 expression level and survival (TCGA dataset). **(A)** Overall survival (GEPIA2) and **(B)** disease-free survival (GEPIA2). The positive results of survival map and Kaplan–Meier curves are listed. GEPIA2: Gene Expression Profiling Interactive Analysis, version 2.

To understand the relationship between PTBP3 expression and prognosis and OS, we divided tumour cases into high- and low-expression groups according to the median expression of PTBP3. TCGA and GEO datasets were used to analyse the differences in survival rates between the two groups. As shown in Figure 2A, the OS rate of the high-expression group was worse in the ACC, LGG, LIHC, LUSC, and PAAD (all *p* < 0.05). DFS analysis data showed that high PTBP3 expression was associated with poor prognosis for ACC, LUSC, and PAAD (Figure 2B). However, the OS and DFS rates of the high-expression group were better in the KIRC group (all *p* < 0.01).

We also used the Kaplan–Meier plotter tool to analyse the relationship between PTBP3 expression and OS, progression-free survival (PFS), first progression and post-progression survival, DFS, disease-specific survival (DSS), distant metastasis-free survival, and relapse-free survival (RFS). As shown in Supplementary Figure S4, the OS rate of the high PTBP3 expression group was poor in LUSC, PAAD, LIHC, and BRCA (all *p* < 0.05). The RFS rate of the high PTBP3 expression group was also worse in the PAAD, LIHC, and BRCA groups (all *p* < 0.05). However, the OS rate of the high PTBP3 expression group was better in the KIRC and STAD groups (all *p* < 0.01).

### Genetic Alteration Analysis Data

**FIGURE 3:**
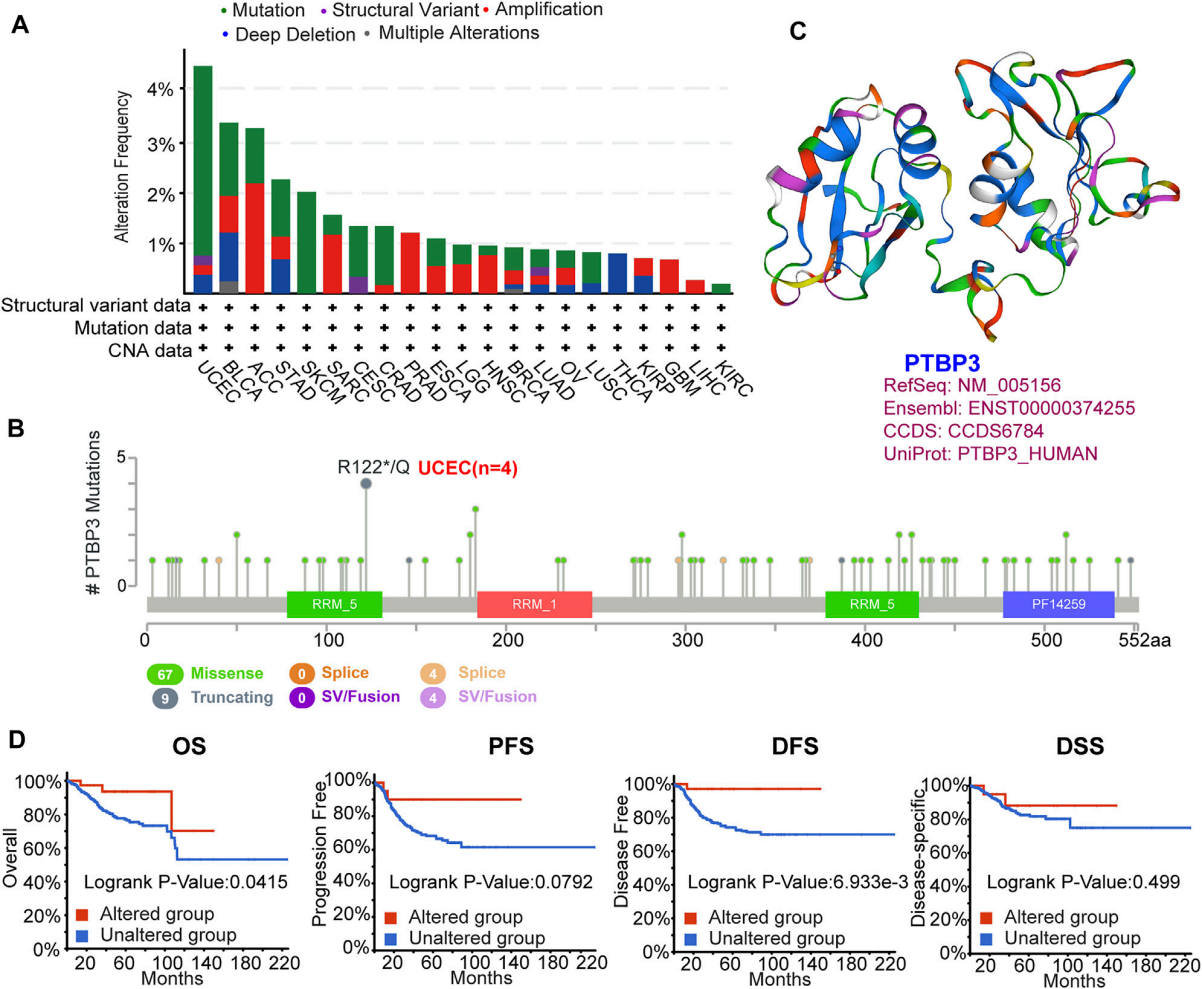
Mutation feature of PTBP3 in human tumours (TCGA dataset). The mutation feature was analysed using the cBioPortal website. **(A)** The alteration frequency with mutation type. **(B)** Mutation site (R122*/Q) within the RRM_5 domain. **(C)** The 3D structure of PTBP3. **(D)** Analysis of the correlation between mutation feature and OS, PFS, DFS and DSS of UCEC. TCGA: The Cancer Genome Atlas; OS: Overall survival; PFS: Progression-free survival; DFS: Disease-free survival; DSS: Disease-specific survival; UCEC: Uterine corpus endometrial carcinoma.

Genetic alterations play important roles in tumorigenesis and development. Therefore, we explored PTBP3 genetic alterations in different tumour tissues. This study indicated that the frequency of PTBP3 alteration (>4%) was the highest in uterine tumours with “mutation” as the primary type. The “amplification” type was the primary type in the ACC, with a frequency of approximately 3% (Figure 3A). Mutations and locations within PTBP3 are shown in Figure 3B. We found that “Missense” was the main type of genetic alteration and that R122*/Q alterations were detected in four cases of UCEC (Figure 3B). Figure 3C shows the 3D structure of the PTBP3 protein. Furthermore, we analysed the association between R122*/Q alterations and prognosis and found that UCEC cases with R122*/Q alterations showed better OS (*p* = 0.0415) and DFS (*p* = 0.0069), but not PFS (*p* = 0.0792) and DSS (*p* = 0.499) (Figure 3D).

We also analysed the relationship between PTBP3 expression and tumour mutational burden (TMB) and microsatellite instability (MSI) in some tumours. PTBP3 expression was positively correlated with TMB in ACC, STAD, PAAD, LUAD, and SARC, but negatively correlated with THYM, UVM, KICH, THCA, and KIRC. Moreover, we observed a positive correlation between PTBP3 expression and MSI for READ, UCEC, STAD, CHOL, and MESO, and a negative correlation for DLBC, HNSC, UCS, PRAD, and ESCA (Supplementary Figure S5).

### Protein Phosphorylation Analysis Data

**FIGURE 4:**
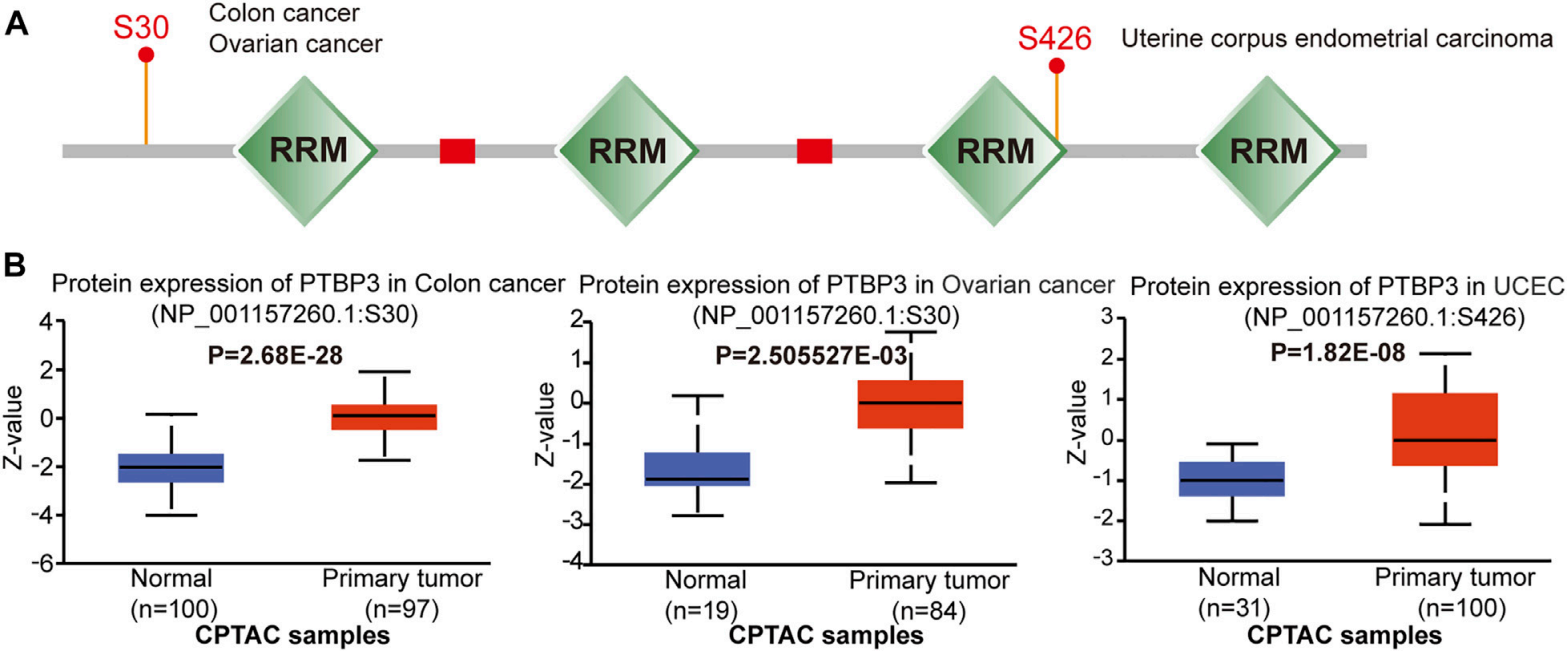
Protein phosphorylation of PTBP3 in different tumours (CPTAC dataset). We analysed the level of PTBP3 phosphoproteins (S30 and S426 sites) between normal and primary tumour tissue in colon cancer, ovarian cancer, and UCEC. **(A)** Phosphoprotein sites of PTBP3 are depicted in a schematic diagram. **(B)** PTBP3 phosphoprotein levels in colon cancer, ovarian cancer, and UCEC. CPTAC: Clinical Proteomic Tumor Analysis Consortium; UCEC: Uterine corpus endometrial carcinoma.

Phosphorylation-dephosphorylation cascades are ubiquitous in tumour development. Thus, we compared PTBP3 phosphorylation levels between normal and tumour tissues. As shown in Figure 4, we found that the S30 locus exhibited a higher phosphorylation level in the primary tumour tissues of colon and ovarian cancers. The S426 locus also exhibited a higher phosphorylation level in the primary tumour tissues of UCEC tissues.

### Immune Infiltration Analysis Data

**FIGURE 5:**
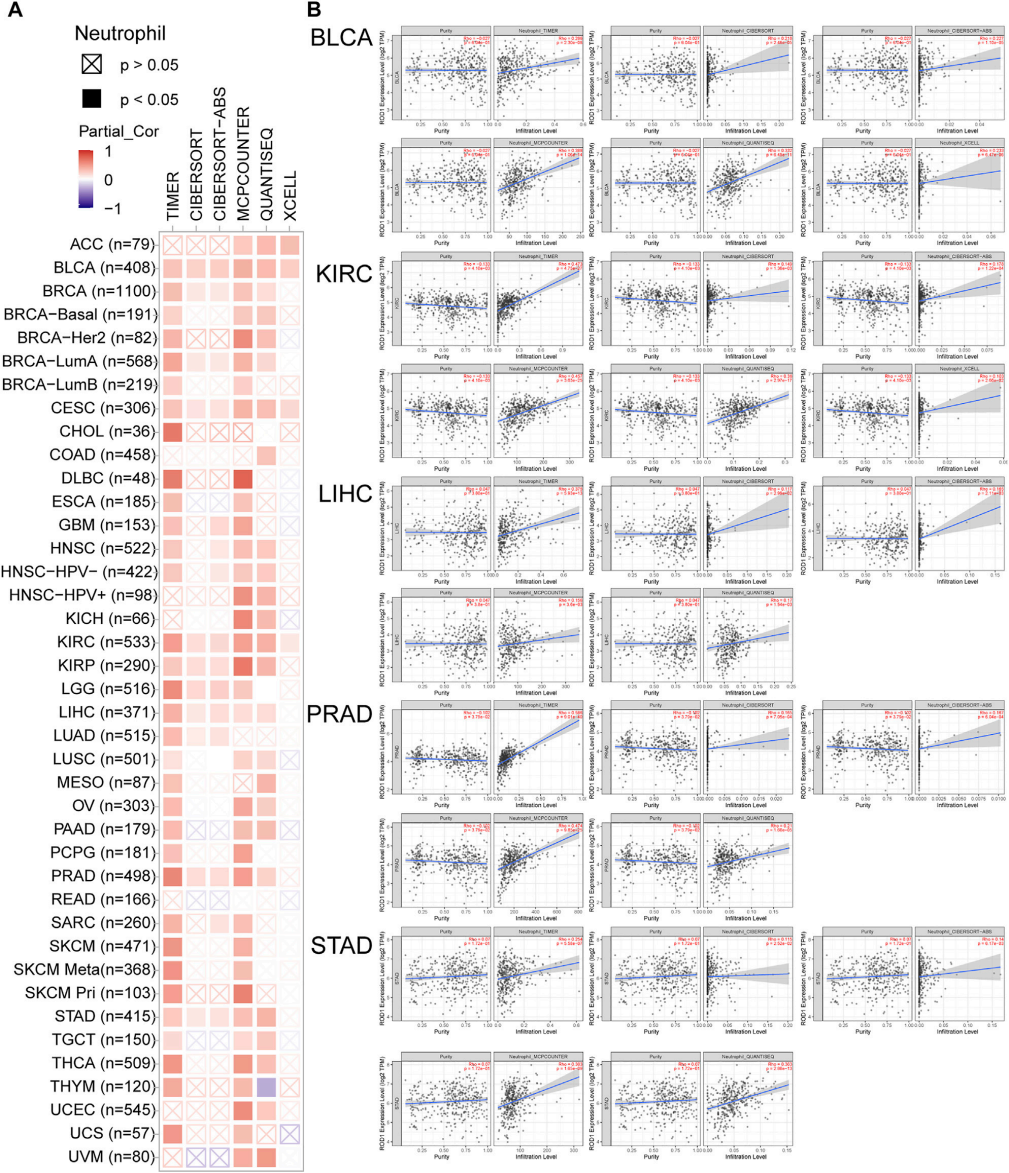
The correlation between PTBP3 expression level and infiltration of neutrophil (TCGA dataset). TIMER, CIBERSORT, MCPCOUNTER, QUANTISEQ and XCELL algorithms were used for the analysis.

PTBP3 mediates alternative splicing regulation of pre-mRNA and plays a role in the regulation of cell proliferation, differentiation, and migration. Tumor-infiltrating immune cells are linked to the initiation, progression, or metastasis of cancer. Thus, the TIMER, CIBERSORT, CIBERSORT-ABS, TIDE, XCELL, MCPCOUNTER, QUANTISEQ, and EPIC algorithms were used to analyse the relationship between PTBP3 expression and tumour-infiltrating immune cells. This study found a positive correlation between PTBP3 expression and neutrophil cell count in most tumour types, especially in BLCA, CESC, DLBC, KIRC, LIHC, PRAD, and STAD. (Figure 5). The relationship between PTBP3 expression and cancer-associated fibroblasts was analysed. We found a positive correlation in most tumour types, especially in ACC, GBM, LIHC, and SARC, while a negative correlation was found in the TGCT tumour (Supplementary Figure S6).

We also analysed the relationship between PTBP3 expression and immune checkpoints (PDCD1, PDCD1LG2, CTLA4, CD274, HAVCR2, LAG3, and TIGIT). In 29 of 33 tumours, the expression of PTBP3 was positively correlated with the expression of immune checkpoint CD274. In 27 of 33 tumours, the expression of PTBP3 was positively correlated with the expression of immune checkpoints PDCD1LG2, while a negative correlation was found in the tumour of THYM. A positive relationship between PTBP3 expression and all immune checkpoints was found in the tumours of UVM, STAD, SKCM, OV, LIHC, and LGG (Supplementary Figure S7).

### N6-Methyladenosine Analysis Data

We also analysed the relationship between PTBP3 expression and N6-methyladenosine (m^6^A) modification. The m^6^A-related genes include ZC3H13, YTHDF1/2/3, YTHDC1/2, RBM15, METTL3/14, KIAA1429, IGF2BP1/2/3, WTAP, HNRNPD, HNRNPC, HNRNPA2B1, FTO, FMR1, ALKBH5 and so on. As shown in Supplementary Figure S8, we found that the expression of most genes was increased in 32 types of tumours, especially in DLBC and UVM.

### Enrichment Analysis of PTBP3-Related Partners

**FIGURE 6:**
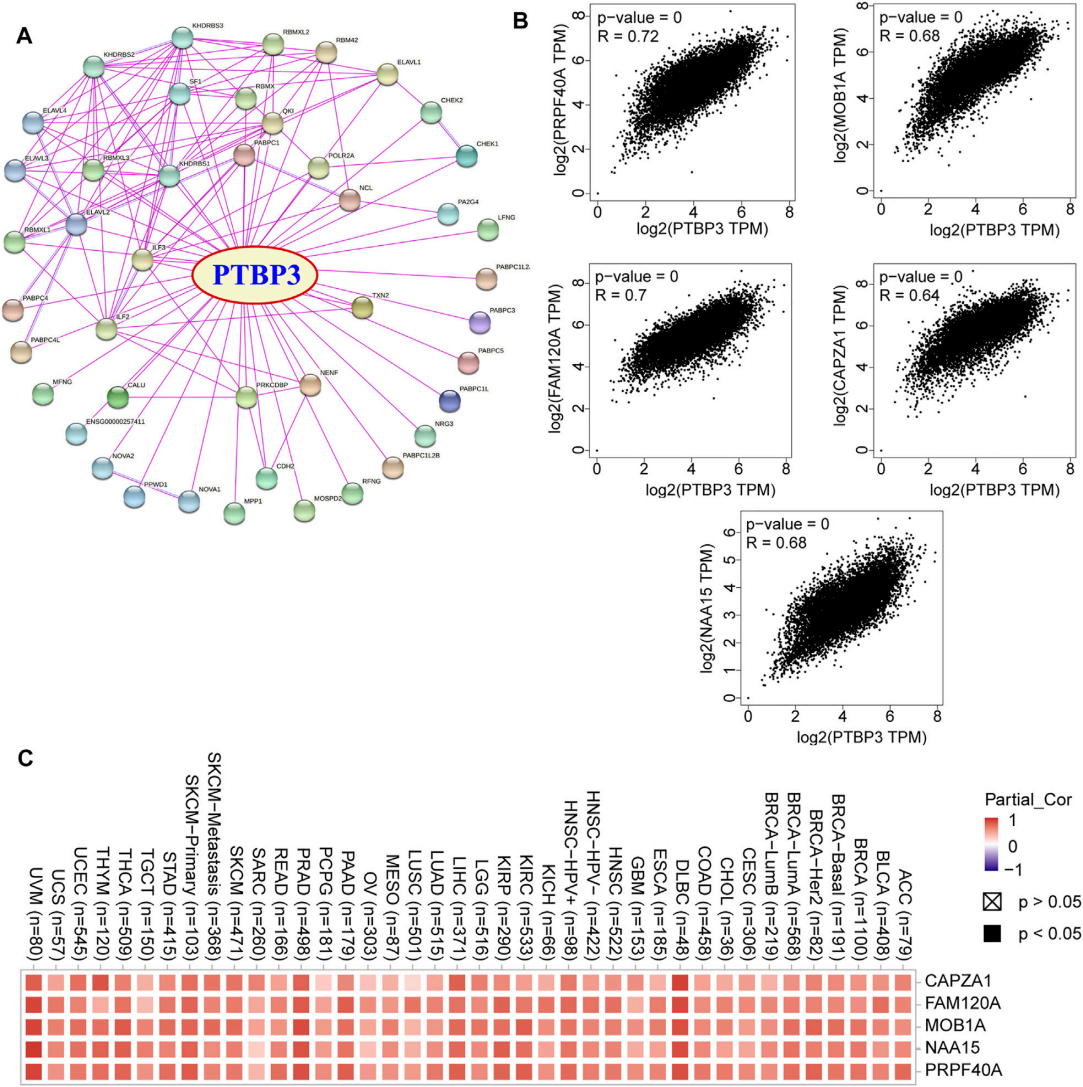
PTBP3-related genes. **(A)** Protein network map of experimentally determined PTBP3-binding proteins (STRING tool). **(B)** Expression correlation between PTBP3 and top 5 TWF1-correlated genes (CAPZA1, FAM120A, MOB1A, NAA15, and PRPF40A) (GEPIA2). **(C)** The heatmap data of PTBP3 and top 5 TWF1-correlated genes in human tumour types. GEPIA2: Gene Expression Profiling Interactive Analysis, version 2.

To further study the molecular mechanism of PTBP3 in tumorigenesis and development, we screened PTBP3-interacting proteins and PTBP3 expression correlated genes for a series of pathway enrichment analyses. We obtained 44 experimentally detected TWF1-binding proteins using the STRING website and the top 100 genes which correlated with PTBP3 expression (Figure 6A). As shown in Figure 6B, the expression of PTBP3 was positively correlated with that of pre-mRNA processing factor 40 homolog A (PRPF40A, R = 0.72), MOB kinase activator 1A (MOB1A, R = 0.68), family with sequence similarity 120A (FAM120A, R = 0.7), capping actin protein of muscle Z-line subunit alpha 1 (CAPZA1, R = 0.64), N-alpha-acetyltransferase 15 (NAA15, R = 0.68) (all *p* < 0.001), and we used these five genes to compute a heatmap that showed their positive association with PTBP3 (Figure 6C).

**FIGURE 7:**
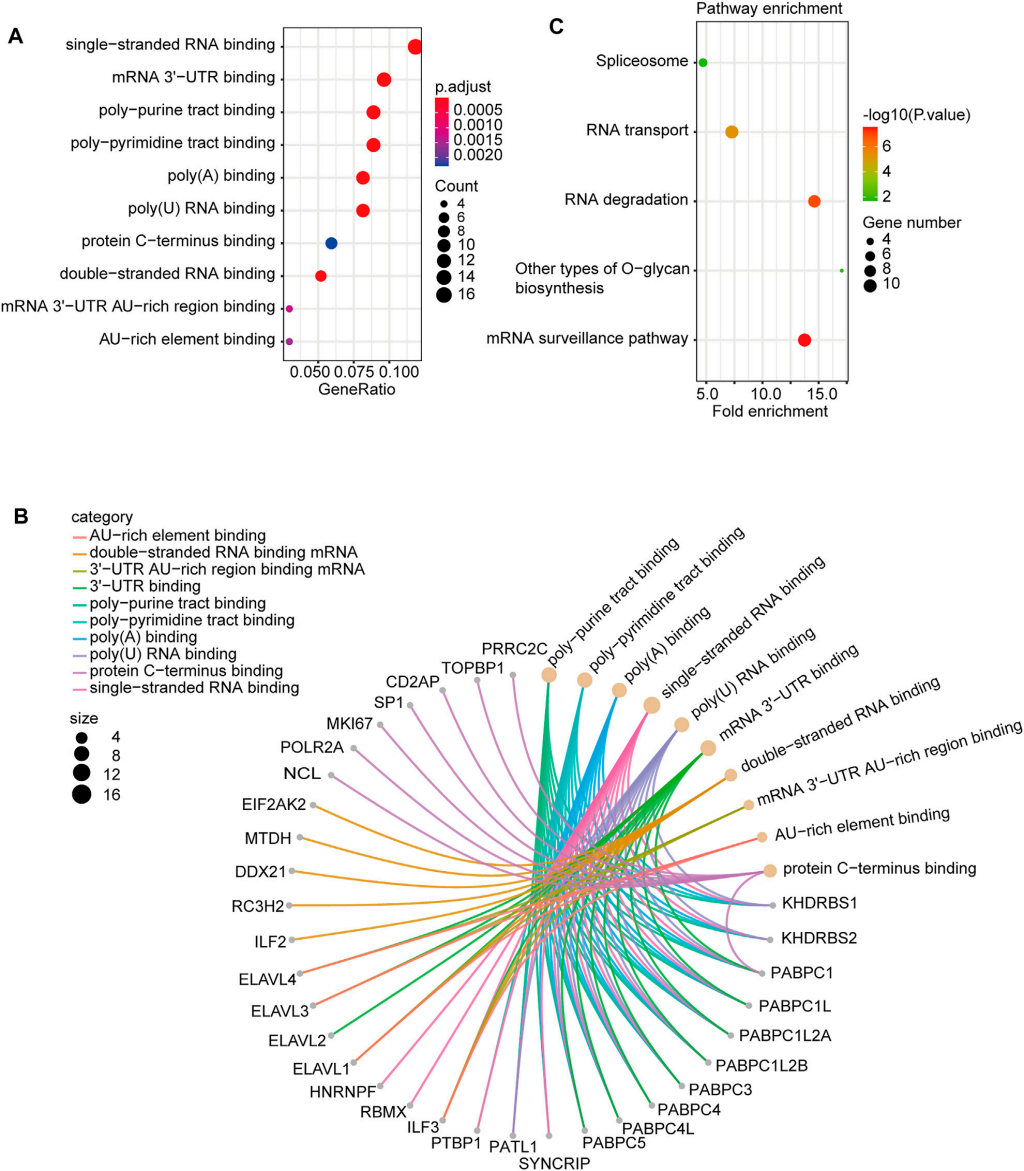
PTBP3-related gene enrichment. **(A)** Dotplot and **(B)** cnetplot of GO analysis. **(C)** KEGG pathway analysis. GO: Gene Ontology; KEGG: Kyoto Encyclopedia of Genes and Genomes.

In this study, we combined the two datasets to perform Gene Ontology (GO) and Kyoto Encyclopedia of Genes and Genomes (KEGG) enrichment analyses. The GO data showed that “single-stranded RNA binding” and “mRNA 3′-UTR binding” were among the top hits. This might be involved in the effect of PTBP3 on tumour pathogenesis (Figure 7A). Figure 7B shows the interaction network of these genes and their molecular functions. KEGG pathway enrichment analysis showed that most genes were related to the “mRNA surveillance pathway” and “RNA degradation” (Figure 7C).

## Discussion

PTBP3, a member of the PTB family, has been reported to be involved in tumorigenesis and progression, such as in lung cancer (Wu et al., 2020), pancreatic cancer (Ma et al., 2020), breast cancer (Hou et al., 2018; Zhou et al., 2018; Liang et al., 2020), gastric cancer (Chen et al., 2014; Liang et al., 2018; Chen et al., 2020), colorectal cancer (Hou et al., 2019), and hepatocellular carcinoma (Yang et al., 2018). However, the oncogenic role of PTBP3 in various human tumours remains unclear. A pan-cancer analysis can identify the difference between normal and tumour tissues, providing a comprehensive understanding of the molecular mechanisms of tumorigenesis and progression (Chakravorty et al., 2019; Chai et al., 2020; Chen et al., 2021; Fiala et al., 2021; Taghvaei et al., 2021). Thus, we performed a pan-cancer analysis of the gene expression profile, survival status, genetic alteration, protein phosphorylation, immune infiltration, N6-methyladenosine, and relevant cellular pathways of PTBP3 in various human tumours based on the TCGA, GEO, and CPTAC databases.

This study showed that PTBP3 is highly expressed in most tumours. The expression levels of PTBP3 in tumours of BRCA, CESC, CHOL, COAD, ESCA, GBM, HNSC, LIHC, LUAD, LUSC, READ, STAD, and UCEC were higher than those in the corresponding adjacent normal tissues, whereas low expression was observed in KICH, KIRC, KIRP, and THCA. Furthermore, we found that PTBP3 overexpression generally predicted poor OS in patients with ACC, LGG, LUSC, and PAAD, in which ACC and LGG were reported for the first time. However, the OS and DFS rates of the high-expression group were better only in the KIRC group. These results suggest that PTBP3 is a potential biomarker for predicting the prognosis of multiple cancers.

Our analysis yielded interesting findings. Especially in N6-methyladenosine analysis, we found that PTBP3 expression is related to all m^6^A markers in almost all human tumours, such as DLBC, PRAD, PAAD, LIHC, KIRP, THYM, and UVM. We speculated that PTBP3 protein is involved in N6-methyladenosine modification, or that it is an important component of methylase. In the immune infiltration analysis, we found that high PTBP3 expression was positively correlated with the cancer-associated fibroblast and neutrophil infiltration levels in most tumours, such as ACC, GBM, LIHC, and SARC. However, its expression was negatively correlated with cancer-associated fibroblasts in TGCT and STAD. In addition, the expression of PTBP3 was positively correlated with the expression of immune checkpoints PDCD1LG2 in most tumours, while a negative correlation was only found in THYM tumours. Our findings may provide a novel clinical biomarker for predicting response to immunotherapy.

A recent study suggested that PTBP3 promotes migration of non-small cell lung cancer (NSCLC) cells by regulating E-cadherin in the epithelial–mesenchymal transition signalling pathway and that there was a significant association between high PTBP3 expression in NSCLC tissues with poor OS (Wu et al., 2020). Moreover, PTBP3 expression is associated with differentiation, lymph node metastasis, and distant metastasis (Wu et al., 2020). However, in this study, we explored LUAD (TCGA, *n* = 515) and LUSC (TCGA, *n* = 501) and found a correlation between the high PTBP3 expression and poor OS prognosis (*p* = 0.0025) and poor DFS (P-0.0048) specific for LUSC but not for LUAD. This was an important discovery, and further research is needed.

Based on the TCGA database, the survival analysis results indicated a correlation between high PTBP3 expression and poor OS and DFS for PAAD. Ma et al. (2020) reported that high PTBP3 expression results in increased resistance to gemcitabine, which is attributed to attenuated autophagy. PTBP3 expression is higher in pancreatic cancer tissues than in corresponding adjacent normal tissues (Ma et al., 2020). The results showed that PTBP3 plays an important role in PAAD.

## Conclusion

This study is the first to report a comprehensive pan-cancer analysis of PTBP3 in human tumours. PTBP3 was highly expressed in most tumours, and predicts poor survival. PTBP3 expression was significant correlated with the immune cell infiltration, TMB, MSI, PDCD1 and m6A-related markers. PTBP3 may be a new and promising prognostic and immunotherapy biomarker in human tumors. We believe that these findings may lay the groundwork for prospective functional experiments and may eventually have a positive impact in clinical practice.

## Data Availability

The original contributions presented in the study are included in the article/Supplementary Material, further inquiries can be directed to the corresponding authors.

## Abbreviations

TCGA, The Cancer Genome Atlas; GTEx, The Genotype-Tissue Expression; TMB, Tumor Mutation Burden; MSI, Microsatellite Instability; ACC, Adrenocortical carcinoma; BLCA, Bladder Urothelial Carcinoma; BRCA, Breast invasive carcinoma; CESC, Cervical squamous cell carcinoma and endocervical adenocarcinoma; CHOL, Cholangiocarcinoma; COAD, Colon adenocarcinoma; DLBC, Lymphoid Neoplasm Diffuse Large B-cell Lymphoma; ESCA, Esophageal carcinoma; GBM, Glioblastoma multiforme; HNSC, Head and Neck squamous cell carcinoma; KICH, Kidney Chromophobe; KIRC, Kidney renal clear cell carcinoma; KIRP, Kidney renal papillary cell carcinoma; LGG, Brain Lower Grade Glioma; LIHC, Liver hepatocellular carcinoma; LUAD, Lung adenocarcinoma; LUSC, Lung squamous cell carcinoma; MESO, Mesothelioma; OV, Ovarian serous cystadenocarcinoma; PAAD, Pancreatic adenocarcinoma; PCPG, Pheochromocytoma and Paraganglioma; PRAD, Prostate adenocarcinoma; READ, Rectum adenocarcinoma; SARC, Sarcoma; SKCM, Skin Cutaneous Melanoma; STAD, Stomach adenocarcinoma; TGCT, Testicular Germ Cell Tumours; THCA, Thyroid carcinoma; THYM, Thymoma; UCEC, Uterine Corpus Endometrial Carcinoma; UCS, Uterine Carcinosarcoma; UVM, Uveal Melanoma.
