# Supplementary material for: New Insights Into PTBP3 in Human Cancers: Immune Cell Infiltration, TMB, MSI, PDCD1 and m6A Markers
Source: Front Pharmacol. 2022 Mar 10;13:811338. doi: 10.3389/fphar.2022.811338 (PMC8960631; doi:10.3389/fphar.2022.811338)
Supplement: Supplementary file 1 [file DataSheet2.docx]

**Dataset sources used in the study**

| **Dataset Sources** | **Location** | **Website** |
| --- | --- | --- |
| TIMER2 | Figure 1A; Figure 2A, B; Figure 5A, B; Figure 6C; Figure S6A, B; Figure S8 | http://timer.cistrome.org/ |
| GEPIA2 | Figure 1B; Figure 6B; Figure 7A, B, C; Figure S3 | http://gepia2.cancer-pku.cn/#index |
| UALCAN | Figure 1C, D; Figure 4A, B | http://ualcan.path.uab.edu |
| cBioPortal | Figure 3A, B, C, D | https://www.cbioportal.org |
| Human Protein Atlas (HPA) | Figure S2A, B; | https://www.proteinatlas.org/ |
| STRING | Figure 6A | https://string-db.org/ |
| GeneCards | Figure S1 | https://www.genecards.org/ |
| Kaplan-Meier Plotter | Figure S4 | http://kmplot.com/analysis/index.php?p=background |
| HOME for Researchers | Figure S5; Figure S7 | https://www.home-for-researchers.com/ |

**R Coding: Figure 7A, B, C**

library("biomaRt")

library("clusterProfiler")

library("org.Hs.eg.db")

library("enrichplot")

library('data.table')

library('dplyr')

library('tibble')

genelist_input <- fread(file=" ",header = T, sep = '\t', data.table = F)

genename <- as.character(genelist_input[,1])

GO_result_MF <- enrichGO(genename, 'org.Hs.eg.db', ont="MF", keyType = "SYMBOL", pvalueCutoff = 0.05)

dotplot(GO_result_MF,showCategory = 20,title = "GO")

cnetplot(GO_result_MF,colorEdge = TRUE,node_lable = "all",circular = TRUE,showCategory = 10)

library(Cairo)

library(stringr)

library(ggplot2)

pathway=read.table(' ',header = T,sep = '\t')

pathway$Term=str_split_fixed(pathway$Term,':',2)[,2]

ggplot(pathway,aes(x=Fold.Enrichment,y=Term))+

geom_point(aes(size=Count,color=-1*log10(PValue)))+

scale_colour_gradient(low='green',high = 'red')+

labs(

color=expression(-log[10](P.value)),

size='Gene number',

x='Fold enrichment',

y='Pathway name',

title = 'Pathway enrichment')+

theme_bw()+

theme(

axis.text.y = element_text(size=rel(1.3)),

axis.title.x = element_text(size=rel(1.3)),

axis.title.y = element_blank()

)
