# Supplementary material for: New Insights Into PTBP3 in Human Cancers: Immune Cell Infiltration, TMB, MSI, PDCD1 and m6A Markers
Source: Front Pharmacol. 2022 Mar 10;13:811338. doi: 10.3389/fphar.2022.811338 (PMC8960631; doi:10.3389/fphar.2022.811338)
Supplement: Supplementary file 2 [file DataSheet1.docx]

Supplementary Material

## Supplementary Figures

**
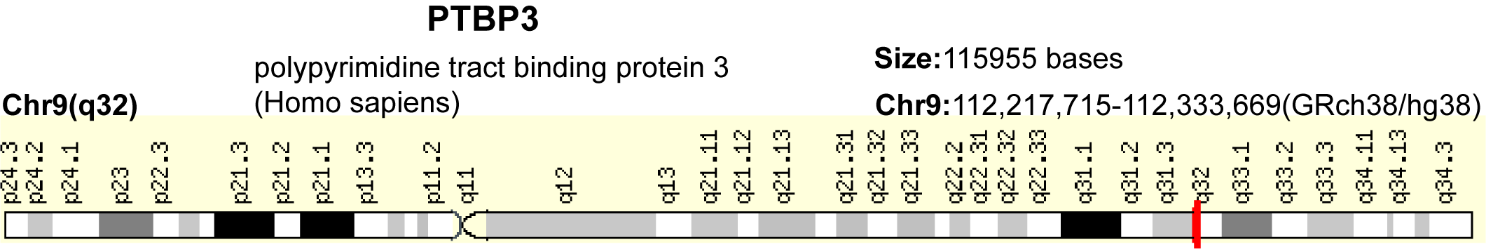
**

**Supplementary Figure 1.** Human PTBP3 gene location in Chr9(q32).

**
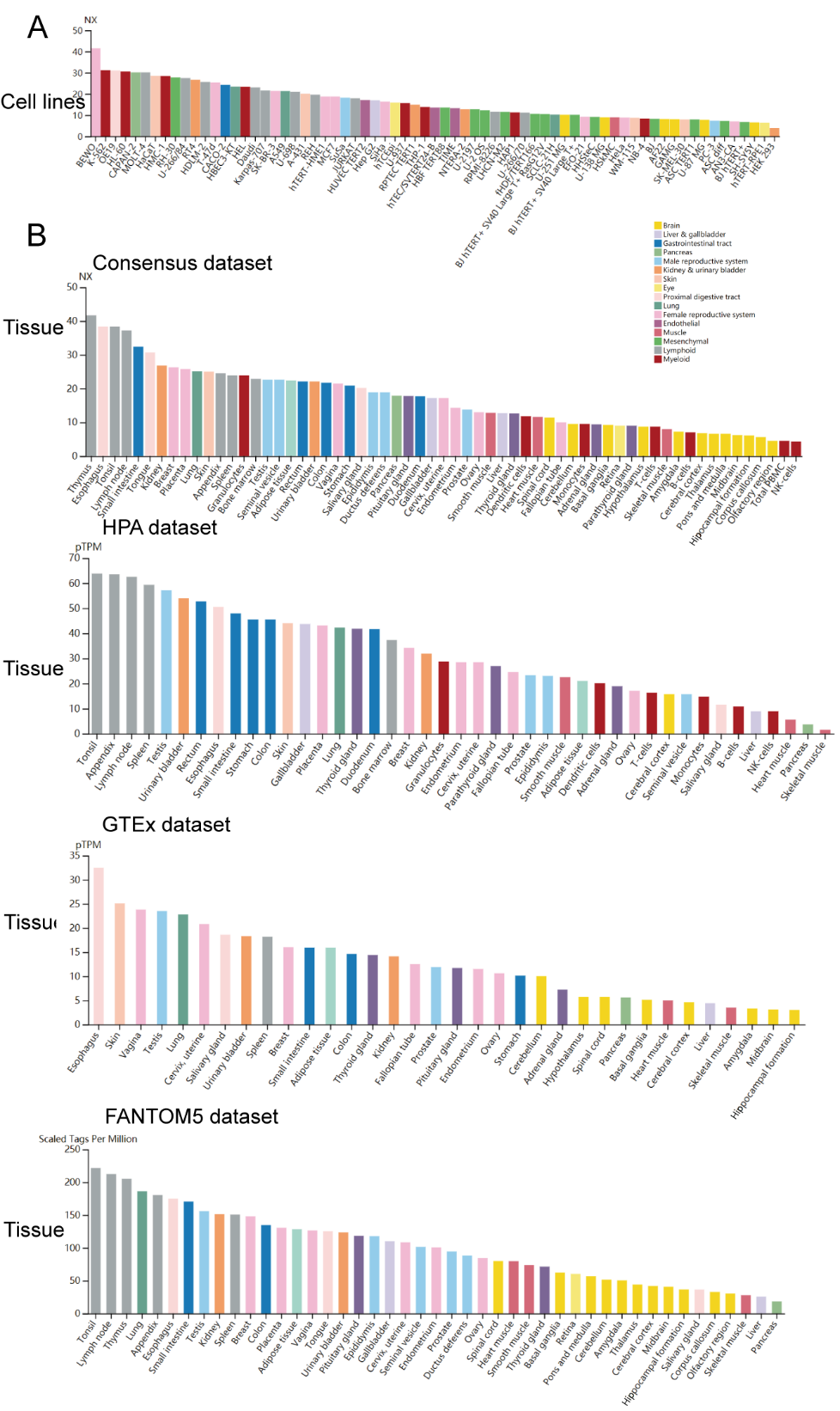
**

**Supplementary Figure 2.** Expression level of PTBP3 in different cell lines (A) and tissues (B) based on Consensus, HPA, GTEx and FANTOM5 dataset.

**
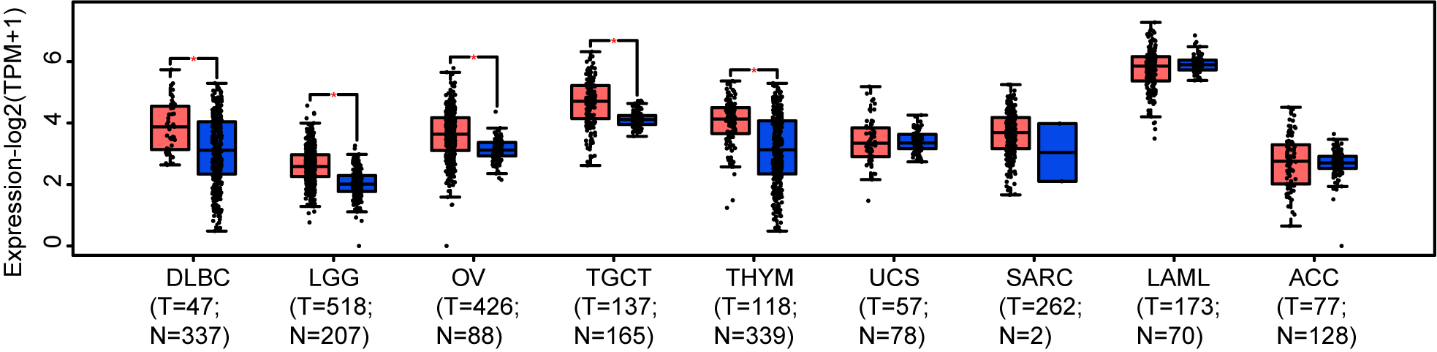
**

**Supplementary Figure 3.** PTBP3 expression level comparison in ACC, BLCA, DLBC, LAML, LGG, OV, PAAD, PCPG, PRAD, SARC, TGCT, THYM and UCS relative to the normal tissues (TCGA+ GTEx dataset).

**
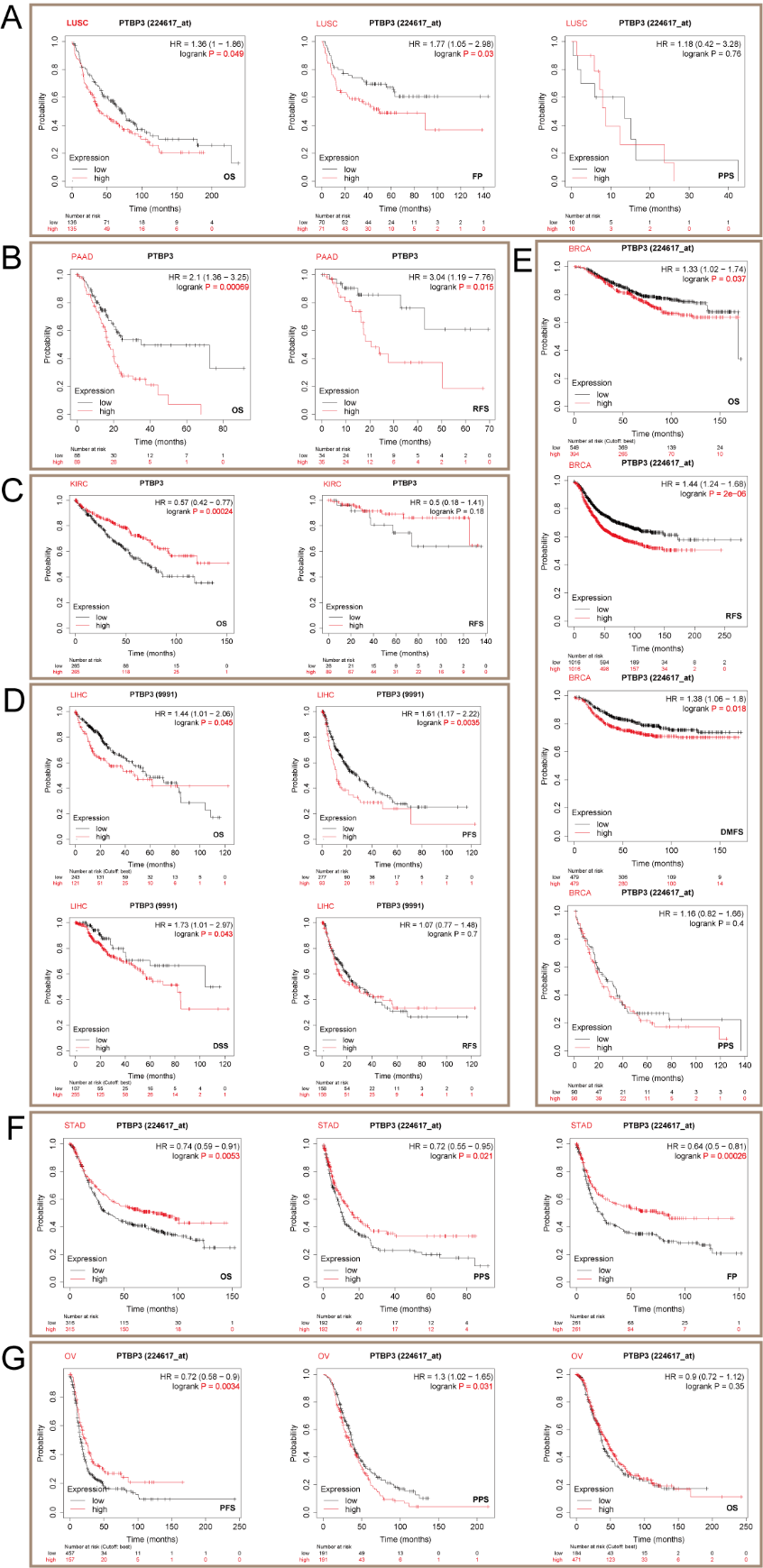
**

**Supplementary Figure 4.** Kaplan-Meier plotter was used to perform the survival analyses (OS, PFS, DMFS, RFS, DSS, FP, and PPS) in LUSC (A), PAAD (B), KIRC (C), LIHC (D), BRCA (E), STAD (F) and OV (G).

**
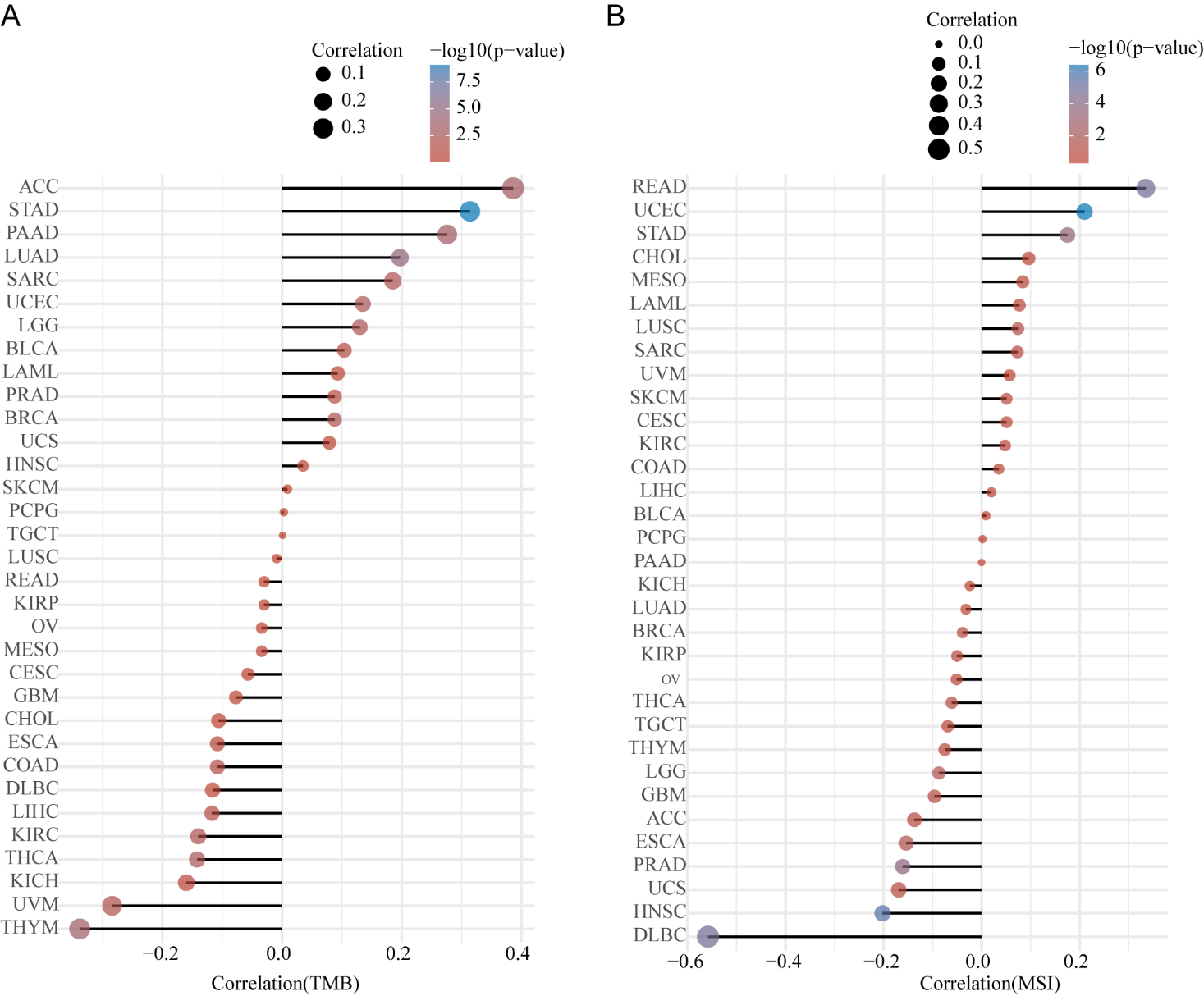
**

**Supplementary Figure 5.** Correlation between PTBP3 expression and tumor mutational burden (TMB) (A) and microsatellite instability (MSI) (B).

**
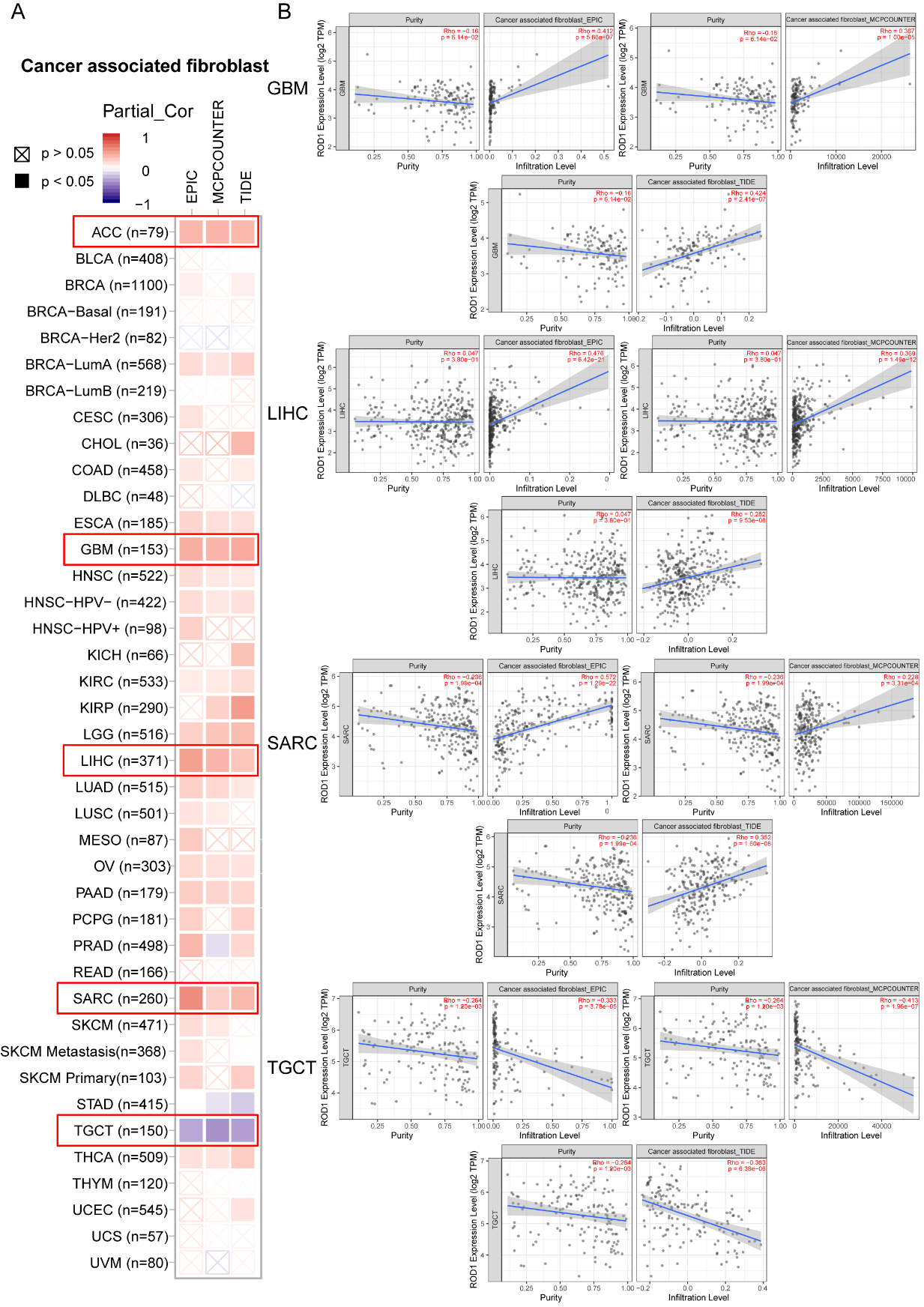
**

**Supplementary Figure 6.** The correlation between PTBP3 expression level and infiltration of cancer associated fibroblasts (TCGA dataset). EPIC, MCPCOUNTER, and TIDE algorithms were used for the analysis. TCGA: The Cancer Genome Atlas.

**
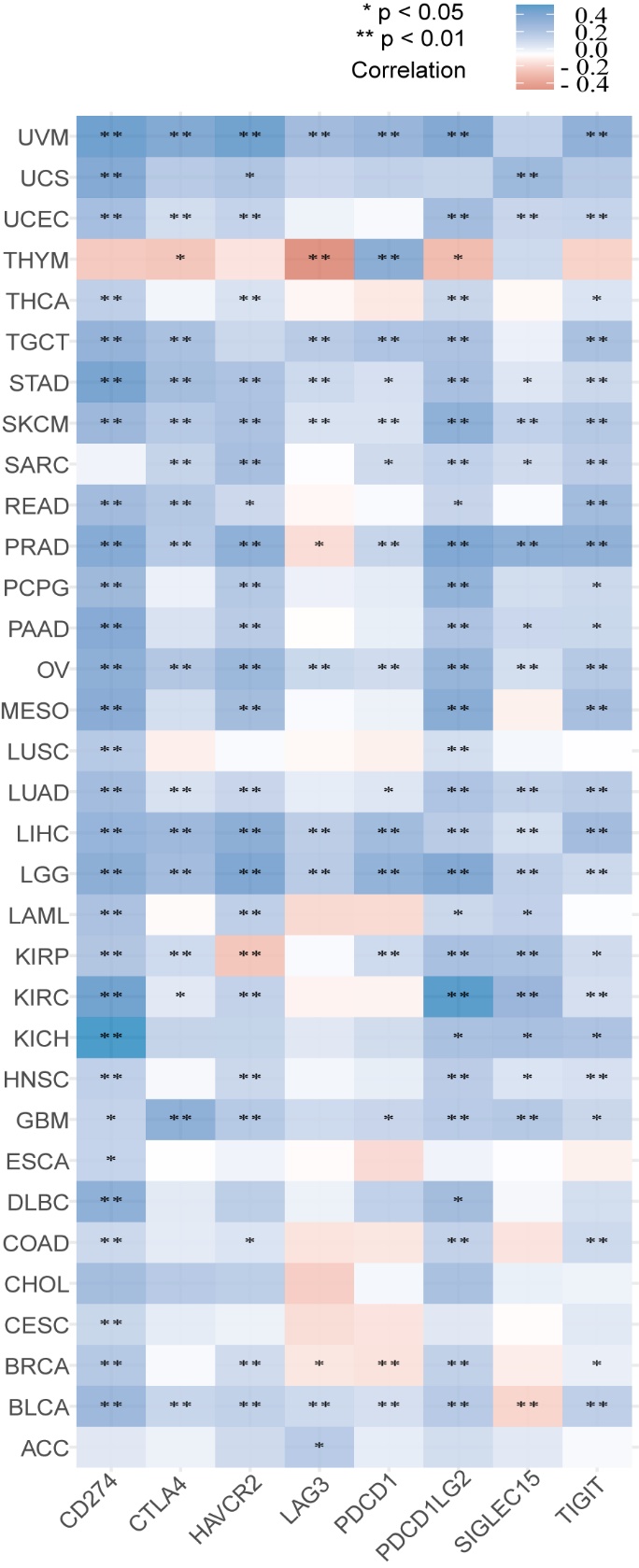
**

**Supplementary Figure 7.** The correlation between PTBP3 expression level and immune checkpoints (PDCD1, PDCD1LG2, CTLA4, CD274, HAVCR2, LAG3, TIGIT) in human tumors (TIMER2).

**
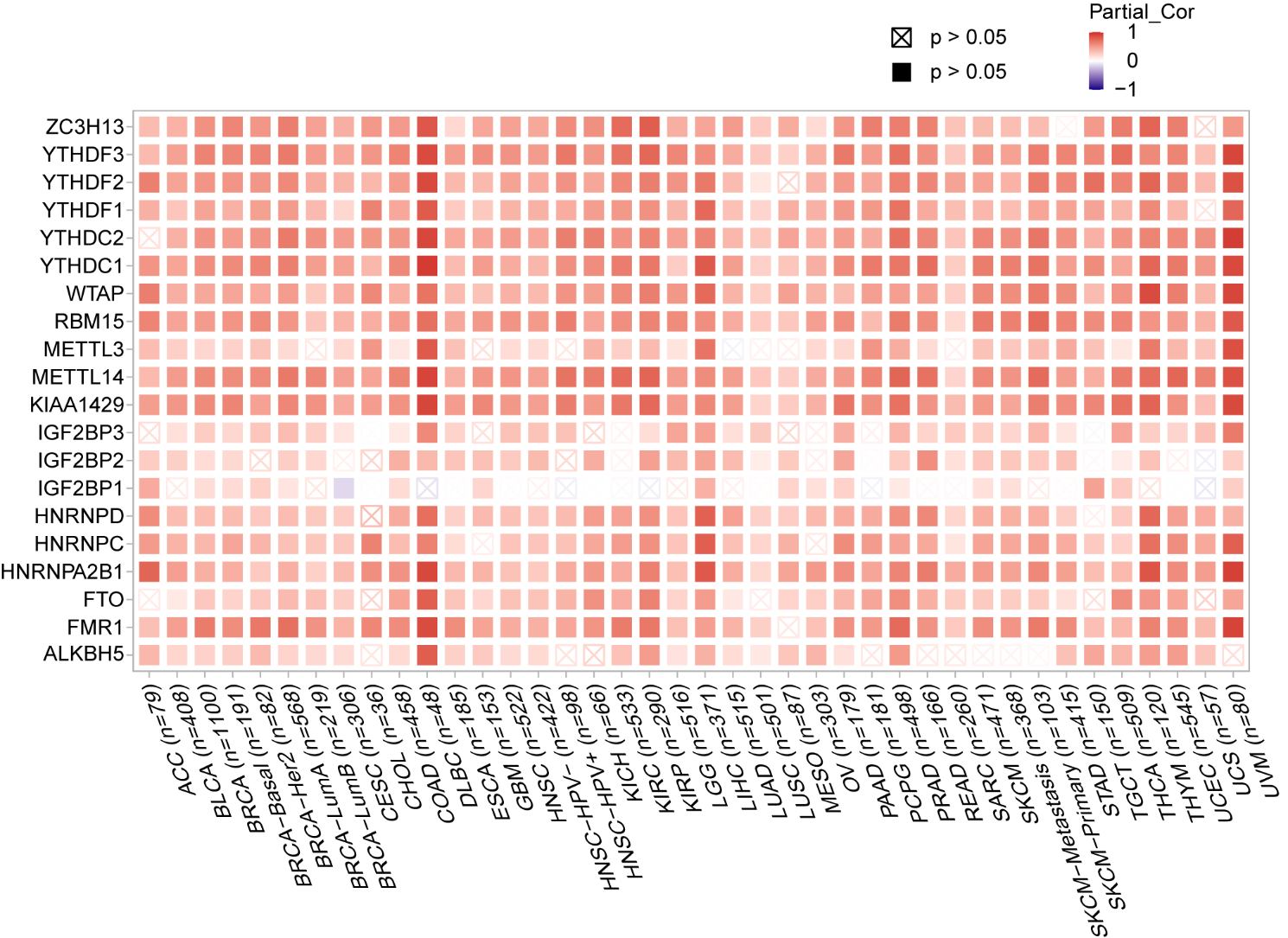
**

**Supplementary Figure 8.** The correlation between PTBP3 expression level and m6A-related genes (PDCD1, PDCD1LG2, CTLA4, CD274, HAVCR2, LAG3, TIGIT) in human tumors (TIMER2).
